# Prevalence of recurrent aphthous stomatitis, oral submucosal fibrosis and oral leukoplakia in doctor/nurse and police officer population

**DOI:** 10.1186/s12903-022-02382-0

**Published:** 2022-08-16

**Authors:** Yundong Liu, Mi He, Tao Yin, Ziran Zheng, Changyun Fang, Shifang Peng

**Affiliations:** 1 Health Management Center, Xiangya Hospital, Central South University, Changsha, 410008 Hunan People’s Republic of China; 2 Department of Stomatology, Xiangya Hospital, Central South University, Changsha, 410008 Hunan People’s Republic of China; 3 Changsha Health Vocational College, Changsha, 410605 Hunan People’s Republic of China; 4 Department of Infectious Diseases, Xiangya Hospital, Central South University, Changsha, 410008 Hunan People’s Republic of China

**Keywords:** Recurrent aphthous stomatitis, Oral submucosal fibrosis, Oral leukoplakia, Oral leukoplakia combined with oral submucosal fibrosis, Doctor/nurse, Police officer

## Abstract

**Background:**

The doctor/nurse and police officer population have some common typical characteristics of great professional pressure and night shift and past studies indicated oral mucosa lesions were closely associated with psychological factors and health-risking behaviors, however the prevalence of recurrent aphthous stomatitis (RAS) and the two commonly seen oral potentially malignant disorders of oral submucosal fibrosis (OSF) and oral leukoplakia in doctor/nurse and police officer in the Betel quid chewing city of Mainland China is unknown The cross-sectional study was to determine the prevalence differences of RAS, oral leukoplakia and OSF among doctor/nurse, police officer and non-doctor/nurse and non-police officer population aged 20–59 years.

**Methods:**

RAS, OSF and oral leukoplakia were examined in doctor/nurse group (male: 659, female: 2439), police officer group (male: 839, female: 262) and non-doctor/nurse and non-police officer group (male: 7576, female: 8129) from 2020-11-01 to 2021-08-31 in Health Management Center, Xiangya Hospital in Changsha city, Hunan province.

**Results:**

The prevalence rates of RAS, OSF, oral leukoplakia and oral leukoplakia combined with OSF in male and female non-doctor/nurse and non-police officer group are 8.32‰ and 10.83‰, 58.08‰ and 1.23‰, 11.75‰ and 0.25‰, 7.66‰ and 0.12‰ respectively. Compared with the non-doctor/nurse and non-police officer population, prevalence rates of RAS in male (24.27‰) and female (20.50‰) doctor/nurse population were significantly higher. Prevalence rates of OSF (21.24‰) and oral leukoplakia (3.03‰) in male doctor/nurse population were significantly less but prevalence rates of OSF (93.71‰), oral leukoplakia (20.17‰) and oral leukoplakia combined with OSF (15.42‰) for male police officer were significantly greater in comparison with male non-doctor/nurse and non-police officer group. OSF and oral leukoplakia prevalence rates were obvious lower for the female than the counterpart male group, but there were no significant differences of OSF and oral leukoplakia prevalence rates between the female non-doctor/nurse and non-police officer and female doctor/nurse group. Oral leukoplakia was not found in the female police officers.

**Conclusions:**

Doctor/nurse population have higher prevalence of RAS. Male doctors/nurses have lower prevalence of OSF and oral leukoplakia, while male police officers have higher prevalence of OSF, oral leukoplakia and oral leukoplakia combined with OSF.

## Introduction

Recurrent aphthous stomatitis (RAS) is characterized with recurrent localized and painful oral ulcers which affect eating, speaking, and even the quality of life [1]. Epidemiological studies show 5%-66% population have RAS indicating this condition is common among young adults all around the world [2–7]. Although the etiology of RAS is unknown, recent studies find sleep disorder [5], late bedtime [8], stress and anxiety [2, 3, 6, 9], no smoking or smoking quitting [3, 4, 10] and family history [7] are the risk factors. Recently a Spanish study found RAS in adolescent population is moderately associated with sleep disorders [5]. A Chinese questionnaire-based survey of university student reveals late bedtime is an independent risk factor for RAS frequency and severity [8]. The US epidemiological report indicates the RAS prevalence is greatest in population aged 17–39 years and never smokers [4]. The doctor and nurse and police officer population have some common typical characteristics of great professional pressure and night shift [11–15]; however the prevalence of RAS in doctor/nurse and in-service police officer population is unknown.

Oral leukoplakia and oral submucosal fibrosis (OSF) are the commonly seen oral potentially malignant disorders (OPMD) [16]. Betel quid chewing, smoking and alcohol use are well accepted causing factors for OSF and oral leukoplakia [9, 17–19]. Betel quid chewing is popular in Taiwan, South and Southeast Asia. In mainland China, Hunan Province is one of the two provinces to produce Betel quid and 16.2% population consume Betel quid [17, 20]. In 2016, betel nuts had been predicted to cause 25,000 cases of oral cancer in Hunan Province [21]. A recent long term follow-up study found 5.6% OSF patients in Mainland China can transform into oral squamous cell carcinoma [22], indicating more attention should be focused on the screening and preventing of OSF. Past studies showed oral leukoplakia is related with the elderly age, smoking, and alcohol intake [9, 23], however, the prevalence of OSF and oral leukoplakia in the doctor/nurse and police officer population in the Betel quid chewing city of Mainland China is unknown.

In order to determine the prevalence rates of RAS, oral leukoplakia and OSF in a betel quid chewing Changsha city in Hunan Province, China and to determine the differences of prevalence rates of RAS, oral leukoplakia and OSF among doctor/nurse, in-service police officer and non-doctor/nurse and non-police officer population, we performed a cross-sectional study in the routine health checkup population in a hospital Health Management Center.

## Materials and methods

### Subjects and data collection

The cross-sectional study was performed from 2020-11-01 to 2021-08-31 in the Health Management Center, Xiangya Hospital, Central South University in Changsha city, Hunan province. The participants received general checkup medically and dentally and were divided into the doctor/nurse group, the police officer group and the non-doctor/nurse and non-police officer group. The doctor/nurse population was aged between 20 and 59 from Xiangya Hospital. The in-service police officer population was aged between 20 and 59 from three districts of Changsha City. The non-doctor/nurse and non-police officer group was aged between 20 and 59, excluding the professional doctor/nurse and police officer and was mainly from general urban residents. The checkup was totally open to the general population and well accepted by citizens both from the economic and convenience perspectives [24]. The total numbers of participants who asked for both medical and oral examination were 4667 for the doctor/nurse group, 1491 for the police officer group and 19,751 for the non-doctor/nurse and non-police officer group while 3098 participants (66.38%) in the doctor/nurse group, 1101 participants (73.84%) in the police officer group and 15,705 participants (79.51%) in the non-doctor/nurse and non-police officer group completed both medical and oral examination and therefore RAS, OSF and oral leukoplakia were clinically examined in the doctor/nurse group (male: n = 659, female: n = 2439), the police officer group (male: n = 839, female: n = 262) and the non-doctor/nurse and non-police officer group (male: n = 7576, female: n = 8129) (Table 1). The reason that participants who did not finish dental exam was mainly because of limited dental chairs. RAS, OSF and oral leukoplakia diagnosis was based on the clinical findings and dental and habit history. The diagnostic criteria for RAS included the presence of recurrent small, round or ovoid ulcers with circumscribed margins, erythematous haloes, and yellow or grey floors in non-keratinized oral mucosa and excluded trauma, drug, tuberculosis and Syphilis associated ulcer and malignant ulcer [1]. The diagnostic criteria for OSF included the presence of palpable fibrous bands or palpable stiffness of a large area and blanching of the mucosa of a large area according to Zhang [17]. The clinical diagnostic criteria for oral leukoplakia followed the guide by Carrard [25]. For atypical or doubted appearance of OSF and oral leukoplakia, the oral mucosa photo records were taken and the diagnosis was determined by advanced clinician and further biopsy. The oral examination was carried out by four dentists with well-trained oral medicine background and there was a high level of intra- and inter-examiner agreement (Kappa value > 0.90) for RAS, OSF and oral leukoplakia among the four dentists. The dental and medical data were managed by the specially designed database software. The test name was omitted and replaced by specific ID and the dental and medical data could be easily retrieved. Informed consents were obtained for all the participants. The study followed the STROBE protocols and all the data collection and procedures associated with this cross-sectional study were approved by Xiangya hospital Ethics Committee (ID: 201503451) and performed according to guidelines.

**Table 1:** Age distribution of the participants who had received both medical and oral examination in the non-doctor/nurse and non-police officer group, the police officer group and the doctor/nurse group

Groups/age range		20–29 years old	30–39 years old	40–49 years old	50–59 years old	Total
Male	Non-doctor/nurse and non-police officer	1484	2164	1831	2097	7576
	Police officer	56	247	252	284	839
	Doctor/nurse	71	384	101	103	659
Female	Non-doctor/nurse and non-police officer	1882	2230	1751	2266	8129
	Police officer	18	84	108	52	262
	Doctor/nurse	551	1359	300	229	2439

### Statistics

The population were divided first into male and female groups and then into the non-doctor/nurse and non-police officer group, the police officer group and the doctor/nurse group. For the different year groups, we then determined the prevalence rates of RAS, OSF, oral leukoplakia and oral leukoplakia combined with OSF of the age specific groups including 20 years old groups (aged 20–29), 30 years old groups (aged 30–39), 40 years old groups (aged 40–49) and 50 years old groups (aged 50–59). The differences of overall prevalence rates between the police officer group and the non-doctor/nurse and non-police officer group and the differences of overall prevalence rates between the doctor/nurse group and the non-doctor/nurse and non-police officer group were compared respectively for males and females with the Chi-Square Test and the odds ratio (OR) and 95% confidence intervals (95% CIs) were then determined. *P* < 0.05 was considered as statistically significant.

## Results

### The prevalence rates of RAS in the non-doctor/nurse and non-police officer group, the police officer group and the doctor/nurse group

**Fig. 1:**
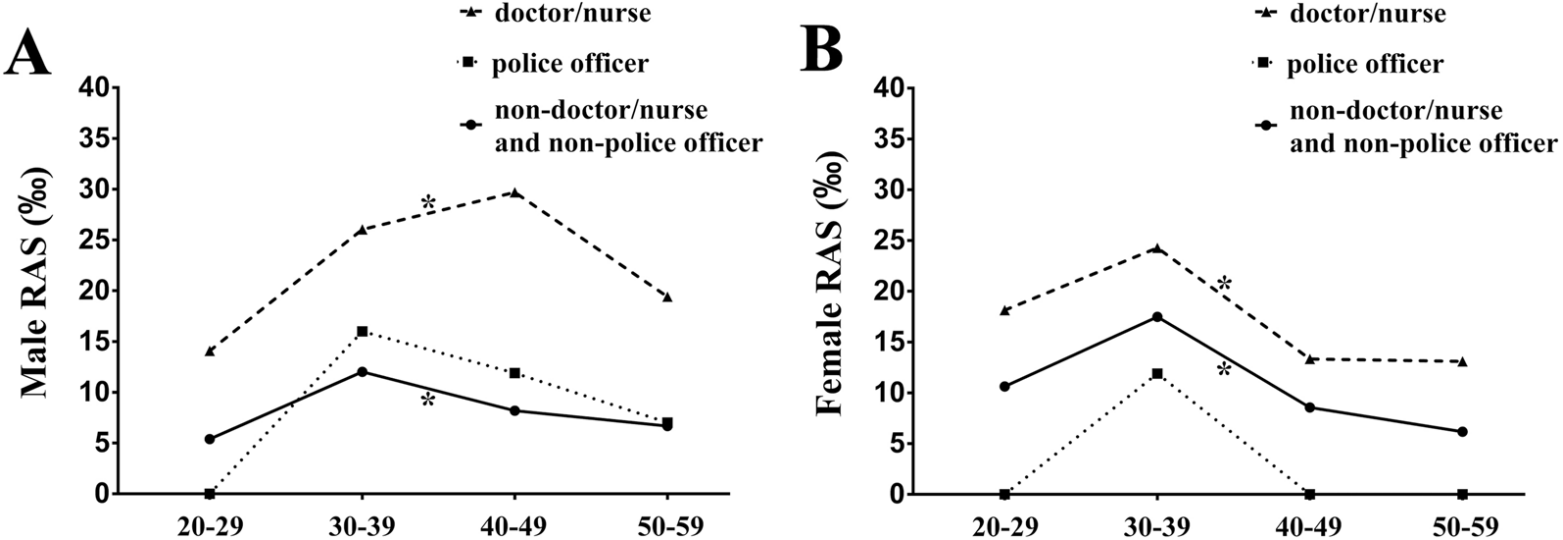
The prevalence rates of RAS in the non-doctor/nurse and non-police officer group, the police officer group and the doctor/nurse group.* indicates statistically significant differences of overall prevalence with *P* < 0.05 between the doctor/nurse group and the non-doctor/nurse and non-police officer goup

The overall prevalence rates of male and female non-doctor/nurse and non-police officer group are 8.32‰ and 10.83‰ respectively (Table 2). Compared with the non-doctor/nurse and non-police officer group, the prevalence rate of RAS is insignificantly greater in the male police officer group (10.68‰) but insignificantly smaller in the female police officer group (3.82‰) (Fig. 1 and Table 2). The overall prevalence rates of male and female doctor/nurse group are 24.27‰ and 20.50‰ and significantly larger than those of the male and female non-doctor/nurse and non-police officer population respectively (Fig. 1 and Table 2).

**Table 2:** The overall prevalence rates of RAS, OSF, oral leukoplakia and oral leukoplakia combined with OSF in the non-doctor/nurse and non-police officer, the police officer and the doctor/nurse population aged 20–59 years

Groups	Gender	Non-doctor/nurse and non-police officer	Police officer			Doctor/nurse		
		Prevalence rate (‰)	Prevalence rate (‰)	OR and 95% CIs (**†)**	*P* value (**†)**	Prevalence rate (‰)	OR and 95% CIs (**‡**)	*P* value (**‡**)
RAS	Male	8.32	10.68	1.29(0.64–2.60)	0.480	24.27	2.97(1.70–5.17)	< 0.001
	Female	10.83	3.82	0.35(0.05–2.52)	0.276	20.50	1.91(1.35–2.71)	< 0.001
OSF	Male	58.08	93.71	1.67(1.31–2.16)	< 0.001	21.24	0.35(0.21–0.60)	< 0.001
	Female	1.23	3.82	3.11(0.40–24.39)	0.255	0.82	0.67(0.15–3.04)	0.598
Oral leukoplakia	Male	11.75	20.17	1.73(1.03–2.92)	0.038	3.03	0.26(0.06–1.04)	0.040
	Female	0.25	0			0.41	1.67(1.15–18.39)	0.673
With both oral leukoplakia and OSF	Male	7.66	15.42	2.03(1.11–3.72)	0.019	0		
	Female	0.12	0			0.41	3.33(0.21–53.32)	0.366

**†** indicates the comparison between the police officer group and the non-doctor/nurse and non-police officer group.

**‡** indicates the comparison between the doctor/nurse group and the non-doctor/nurse and non-police officer group.

### The prevalence rates of OSF in the non-doctor/nurse and non-police officer group, the police officer group and the doctor/nurse group

**Fig. 2:**
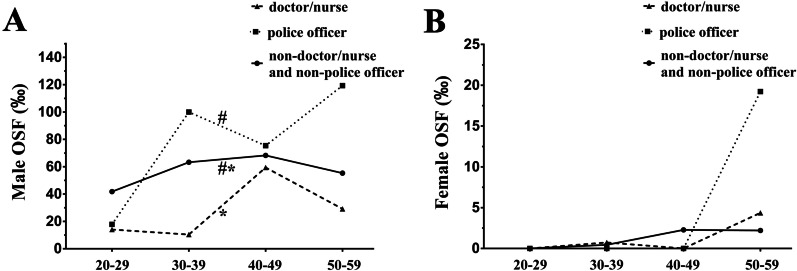
The prevalence rates of OSF in the non-doctor/nurse and non-police officer group, the police officer group and the doctor/nurse group. * indicates statistically significant differences of overall prevalence with *P* < 0.05 between the male doctor/nurse group and the male non-doctor/nurse and non-police officer group. # indicates statistically significant differences of overall prevalence with *P* < 0.05 between the male police officer group and the male non-doctor/nurse and non-police officer group

The overall prevalence rates of OSF in the male and female non-doctor/nurse and non-police officer group are 58.08‰ and 1.23‰ respectively and there was a significant difference between the two groups (Table 2). Compared with the male non-doctor/nurse and non-police officer groups, the overall prevalence rate of OSF was significantly greater in the male police officer group (93.71‰) while the overall prevalence rate of OSF in the male doctor/nurse group (21.24‰) was significantly smaller (Fig. 2A and Table 2). There was no significant difference of overall prevalence rates of OSF between the female doctor/nurse group or the female police officer and the female non-doctor/nurse and non-police officer population (Fig. 2B and Table 2).

### The prevalence rates of oral leukoplakia and oral leukoplakia combined with OSF in the non-doctor/nurse and non-police officer group, the police officer group and the doctor/nurse group

**Fig. 3:**
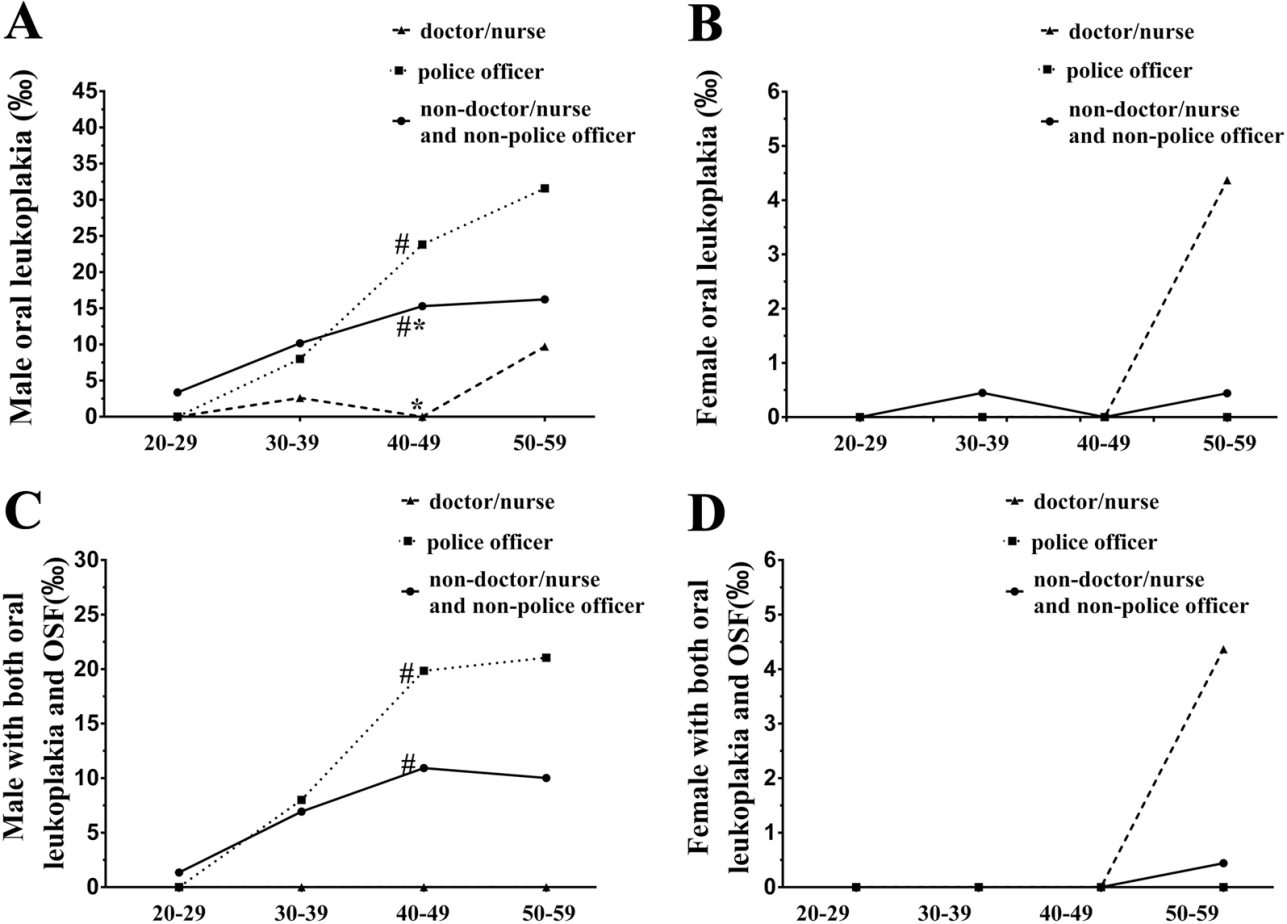
The prevalence rates of oral leukoplakia and oral leukoplakia combined with OSF in the non-doctor/nurse and non-police officer group, the police officer group and the doctor/nurse group. * indicates statistically significant differences of overall prevalence with *P* < 0.05 between the male doctor/nurse group and the male non-doctor/nurse and non-police officer group. # indicates statistically significant differences of overall prevalence with *P* < 0.05 between the male police officer group and the male non-doctor/nurse and non-police officer group

The overall prevalence rate of oral leukoplakia in the male and female non-doctor/nurse and non-police officer group are 11.75‰ and 0.25‰ respectively (Fig. 3A and Table 2). The overall prevalence of oral leukoplakia (20.17‰) in the male police officer group was significantly greater while the overall prevalence rate of oral leukoplakia (3.03‰) in the male doctor/nurse group was significantly smaller in comparison with the male non-doctor/nurse and non-police officer group (Fig. 3A and Table 2). The overall prevalence rate of oral leukoplakia in the female non-doctor/nurse and non-police officer group was significantly lower than that of male non-doctor/nurse and non-police officer group (Fig. 3 and Table 2). Oral leukoplakia was not found in the female police officer group (Fig. 3B and Table 2). There was no significant difference of overall prevalence rates of oral leukoplakia between the female non-doctor/nurse and non-police officer group and the female doctor/nurse group (Fig. 3B and Table 2).

Further, we analyzed the prevalence rates of participants with both oral leukoplakia and OSF. The overall prevalence rates of oral leukoplakia combined with OSF in the male and female non-doctor/nurse and non-police officer group are 7.66‰ and 0.12‰ respectively (Table 2). Compared with the male non-doctor/nurse and non-police officer group, the overall prevalence rate of oral leukoplakia combined with OSF (15.42‰) in the male police officer group was significantly greater while oral leukoplakia combined with OSF was not found in the male doctor/nurse group (Fig. 3C and Table 2). The overall prevalence rates of oral leukoplakia combined with OSF in female non-doctor/nurse and non-police officer group and female doctor/nurse are 0.12‰ and 0.41‰ respectively without significant difference (Fig. 3D and Table 2). Oral leukoplakia combined with OSF was not found in the female police officer group (Fig. 3D and Table 2).

## Discussion

To determine the differences of prevalence rates of RAS, oral leukoplakia and OSF among doctor/nurse, in-service police officer and non-doctor/nurse and non-police officer population, we performed the cross-sectional study in the health checkup population in a betel quid chewing Mainland China city. We found the prevalence rates of RAS, OSF, oral leukoplakia and oral leukoplakia combined with OSF in male and female non-doctor/nurse and non-police officer group are 8.32‰ and 10.83‰, 58.08‰ and 1.23‰, 11.75‰ and 0.25‰, 7.66‰ and 0.12‰ respectively. Compared with non-doctor/nurse and non-police officer, prevalence rates of RAS in male (24.27‰) and female (20.50‰) doctor/nurse population were significantly larger. Prevalence rates of male doctor/nurse OSF (21.24‰) and oral leukoplakia (3.03‰) were significantly less and oral leukoplakia combined with OSF was not found in the male doctor/nurse population but prevalence rates of OSF (93.71‰), oral leukoplakia (20.17‰) and oral leukoplakia combined with OSF (15.42‰) for male police officer were significantly greater in comparison with male non-doctor/nurse and non-police officer group. OSF and oral leukoplakia prevalence rates were obvious lower for the female than the counterpart male group, but there were no significant differences of OSF and oral leukoplakia prevalence rates between the female non-doctor/nurse and non-police officer and female doctor/nurse group. Oral leukoplakia and oral leukoplakia combined with OSF were not found in the female police officer group.

The oral examination was only one part of the regular health examination [24], so we could exclude the sample including bias and the sample could reflect the urban general residents, doctor/nurse population and police officers. The prevalence of RAS peeks at 30 years population, which is in agreement with most studies that indicated young adults have greatest prevalence of RAS [3, 4]. The prevalence rates of OSF, oral leukoplakia and oral leukoplakia combined with OSF increase with age and peek at 40–50 years group for the male non-doctor/nurse and non-police officer population. The overall prevalence of OSF is similar with that found in a Taiwan investigation [18]. In comparison with the community investigation in Shanghai [23], the prevalence of RAS in non-doctor/nurse and non-police officer group is slightly lower while the prevalence of male oral leukoplakia (11.75‰) in Hunan is obvious higher than that of oral leukoplakia (2.2‰) in Shanghai, mainly due to the OSF related oral leukoplakia (male: 7.66‰) in Betel quid chewing Hunan Province. All these data indicate the reliability of the investigation data.

For the doctor/nurse population, the prevalence rates of both male and female RAS are significantly greater, while the prevalence rate of OSF and oral leukoplakia in male doctor/nurse population is significantly smaller when compared with the male non-doctor/nurse and non-police officer group. The RAS prevalence rates for female and male doctor/nurse population are also greater than that found in the community population in Shanghai (1.68%). There might be three combined factors for the increased RAS in doctor/nurse population. First, recent researches indicate the doctor and nurse as one of the population with greatest professional pressure suffers from more distress [26] and RAS is associated with psychological factors [2, 3, 6]. Long-term follow up study from university to career also found the RAS prevalence rate is relatively high in the doctor/nurse and dental profession [2, 7, 27]. Second the doctor/nurse population in the hospital must take regular night shifts. Past studies indicate physicians often sleep less [13] and RAS is related with poor sleepy quality [5] and even late bedtime [8]. Last, Chinese regulation bans smoking in hospital and hospital is smoking free facility. In addition to the medical background and well-known harms of smoking, the doctor/nurse population in China might smoke less in contrary to the past study [13] and less smoking and smoking quitting increase the prevalence of RAS [3, 4, 10]. In contrary the male doctor/nurse population have significantly less prevalence rates of OSF and oral leukoplakia and oral leukoplakia combined with OSF was not found in the male doctor/nurse population. Betel quid chewing and smoking are closely associated with OSF and oral leukoplakia [17, 18]. The obvious lower level of prevalence rates of OSF and oral leukoplakia in female population might explained by the lower rates of Betel quid chewing and smoking habits for females in comparison with males. Although substance addiction is found in a previous investigation of physician population [13] and Betel quid chewing are common among males in Hunan province [17], the OSF and oral leukoplakia are relatively lower in the doctor/nurse population partly because of the well medical knowledge of the Betel quid chewing and smoking associated disorders, especially the high Betel quid associated oral cancer in Hunan province [21] and the Chinese regulation forbidding smoking in hospital.

For the police officer population, we found the male police officer population have significantly highest level of prevalence of OSF, oral leukoplakia and oral leukoplakia combined with OSF and insignificant greater prevalence rates of RAS while the female police officer population have the insignificantly lowest level of RAS and oral leukoplakia and oral leukoplakia combined with OSF were not found in the female police officer group. There might be three main reasons for the increased OSF and oral leukoplakia in the male police officer population. First the male police officers often face higher levels of occupational anxiety and depression [11, 12, 14, 15, 28]. In Hunan Province, China, 16.2% population consume Betel quid [17]. OSF prevalence rate is 93.71‰ in male police population, obviously greater than the previous report in general population [17], indicating a greater proportion of male police officers chew betel quid to relief psychological pressure. Second. The past study showed the betel quid chewer often have the common habit of smoking and drinking [17, 18]. More evidences suggest the habit of betel quid chewing as well as smoking and alcohol use is also addicted [29]. The addiction mechanism of betel quid chewing, smoking and alcohol may increase the difficulty in quitting [28]. Third although both the male police officer and the doctor/nurse population may take night shift, the policeman may chew betel quid and smoke to keep refresh as a result of lack of deep knowledge of the medical harms brought by these behaviors compared with the medical professionals. Smoking is negatively associated with RAS and the RAS in the male police officer is only slightly elevated compare with the general population. For the female police officer, the level of prevalence of RAS is relatively lower and oral leukoplakia is not found. Recent study in China showed the oral mucosal disorder is closely associated with psychological factors [9]. In contrary to Canadian police officers [30], where policewomen were reported to have greater mental disorder rates relative to the general population, policewomen in China mainly perform general affairs and might have lower psychological pressure so that they have less oral mucosal diseases.

In conclusion, both the male and female doctor/nurse population have relatively higher prevalence rates of RAS and male doctors/nurses have relatively lower prevalence rates of OSF and oral leukoplakia, so that interventions to relief stress and drug development to alleviate RAS are needed. The male police officer population have relatively higher prevalence of OSF, oral leukoplakia and oral leukoplakia combined with OSF. OSF and oral leukoplakia are well known risk factors for malignant transformation, so that medical education and policies to decrease stress and to quit the harmful habits of betel quid chewing and smoking are need.

## Data Availability

All data generated or analyzed during this study are included in this article.
